# Association between vertebral endplate lesions and sagittal spinal alignment: a retrospective imaging analysis in a selected adult patient population

**DOI:** 10.1186/s41747-026-00746-7

**Published:** 2026-06-05

**Authors:** Tito Bassani, Riccardo Cecchinato, Sara Pasi, Maria Ludovica Pallotta, Luca Maria Sconfienza, Domenico Albano, Marco Brayda-Bruno

**Affiliations:** 1 IRCCS Istituto Ortopedico Galeazzi, Milan, Italy; 2 Department of Biomedical Sciences for Health, University of Milan, Milan, Italy; 3 Department of Radiology, ASST Grande Ospedale Metropolitano Niguarda, Milan, Italy; 4 Dipartimento di Scienze Biomediche, Chirurgiche ed Odontoiatriche, Università Degli Studi di Milano, Milano, Italy

**Keywords:** Lordosis, Lumbar vertebrae, Magnetic resonance imaging, Radiography, Spine

## Abstract

**Objective:**

Most endplate lesions are asymptomatic and incidentally detected. Prevalence in adults ranges from 28% to 46%, but their clinical relevance remains unclear. A knowledge gap persists regarding how spinal alignment parameters affect the type and distribution of lesions across lumbar levels. This study aims to bridge the gap by examining associations between alignment variables and lesion indices.

**Materials and methods:**

A retrospective study was conducted on 584 adults who underwent spine magnetic resonance imaging (MRI) and standing radiographs within 6 months, with at least one spinal level exhibiting an endplate lesion between T12–L1 and L5–S1. Lesions were graded using a validated MRI-based classification system (normal, wavy/irregular, notched, Schmorl nodes). Modic changes, sagittal alignment parameters, and lumbopelvic profile (Roussouly classification) were also evaluated. Associations between demographic and alignment variables with lesion indices were analyzed through correlation and regression models.

**Results:**

Endplate lesions were found in 60% of spinal levels (30% wavy/irregular, 20% notched, 10% Schmorl nodes). Modic changes were absent in 84% of levels, yet 53% of these displayed lesions. Lesion severity increased with age and was slightly higher in males. Reduced lumbar lordosis correlated inversely with lesion indices, indicating a higher prevalence in flatter alignments. Severe lesions were somewhat more common in upper lumbar levels. Lumbopelvic profiles 1 and 2 showed higher lesion prevalence and severity, though profile type was not an independent predictor.

**Conclusion:**

Endplate lesions increase with age and reduced lumbar lordosis. While sagittal parameters and lumbopelvic profiles modulate lesion distribution, their predictive power is limited, supporting a multifactorial etiology of endplate degeneration.

**Relevant statement:**

Endplate lesions increase with age and reduced lumbar lordosis. Although sagittal alignment and lumbopelvic profiles influence their distribution, these parameters show limited predictive value, highlighting the multifactorial nature of endplate degeneration and the need for integrated morphological and biomechanical assessment in spinal evaluation.

**Key Points:**

- Endplate lesions prevalence and severity increase with age, occur slightly more in upper lumbar levels, and show a weak-to-moderate Modic changes association.
- Reduced lumbar lordosis could predispose to endplate lesions, suggesting unfavorable load transfer across spinal segments.
- Lumbopelvic profile influences lesion distribution but is not an independent predictor of lesion presence or type.

**Graphical Abstract:**

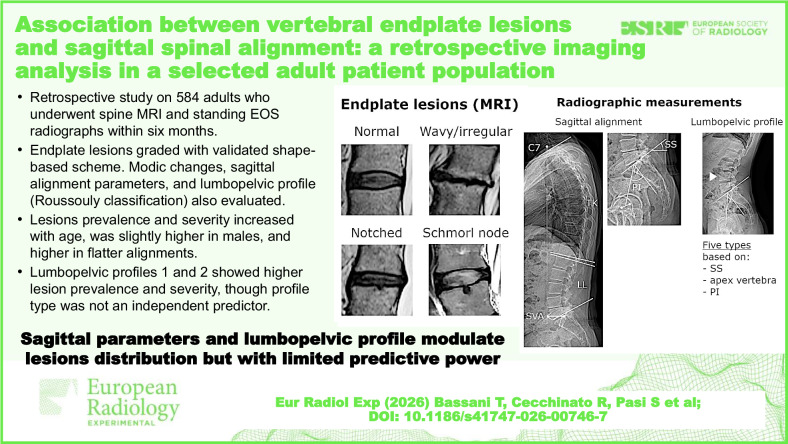

## Background

Endplate lesions involve both the cartilaginous and bony components of the vertebral endplates and are typically evaluated using magnetic resonance imaging (MRI). Although most endplate lesions are asymptomatic and detected incidentally [1], their prevalence in the adult population has been reported to range from 28% to 46%, depending on the imaging modality and classification criteria applied [2]. Despite their frequent occurrence, the clinical significance of endplate lesions remains uncertain. This reflects the terminology of endplate ‘lesions’ as defined in standard medical and pathological contexts, referring broadly to any structural or morphological alteration in tissue—regardless of etiology or clinical manifestation. This definition encompasses abnormalities detected through imaging, even in the absence of clinical symptoms, and has led many authors to adopt the term ‘lesion’ in previous studies [1–9], alongside related terms such as ‘defects’ or ‘damage’ [10–12]. Beyond these considerations, prior studies suggest that their occurrence and progression represent a cumulative process, with higher lesion grades more commonly observed in older individuals of both sexes [6, 7].

To facilitate standardized assessment, several classification systems have been proposed [5, 10]. Among these, the scheme introduced by Brayda-Bruno et al in 2018 has gained wide acceptance owing to its simplicity and reproducibility in clinical practice [4, 5]. This MRI-based system categorizes endplate morphology into four principal types: normal, wavy/irregular, notched, and Schmorl node. It has demonstrated high inter- and intra-observer reliability (Fleiss’ κ values of 0.73 and 0.89, respectively) and has been applied in a large-scale study involving nearly 1,000 patients with non-specific low back pain [5].

Endplate lesions have been associated with intervertebral disc degeneration and Modic changes, although the available evidence remains limited and partially inconsistent. Several studies have reported significant associations, suggesting a shared degenerative pathway [8, 12, 13]. However, the clinical implications of these associations remain unclear. Notably, our previous work found no significant relationship between endplate lesions and patient-reported outcomes such as pain intensity or disability scores in individuals with non-specific low back pain [3].

Beyond localized disc and vertebral pathology, sagittal spinal alignment may influence both the development and distribution of endplate lesions. Lumbar sagittal alignment, particularly in the lumbosacral region, governs load transmission through the vertebral column and may predispose certain spinal levels to mechanical stress and subsequent lesion formation. The Roussouly classification provides a widely accepted framework for characterizing sagittal alignment on standing radiographs. It distinguishes five lumbopelvic profiles based on sacral slope, pelvic incidence, and lumbar curvature, thereby offering insights into biomechanical loading patterns across the spine [14, 15].

A critical knowledge gap persists regarding the impact of spinal alignment parameters on the type and distribution of endplate lesions across lumbar levels. Clarifying this relationship has important clinical implications, as it could enhance diagnostic precision and inform individualized management strategies—ranging from conservative rehabilitation approaches to surgical procedures designed to restore optimal spinal alignment. While a priori hypotheses are difficult to advance, differences in the prevalence and distribution of lesion types according to lumbopelvic profile and sagittal parameters are plausible. Morphological injuries at the intervertebral disc level in degenerative spinal conditions may result from alterations in load transfer patterns and shearing forces, which are influenced by spinal alignment [16–18]. Accordingly, the aim of the present study is to investigate the association between vertebral endplate lesions and sagittal spinal alignment in a selected adult orthopedic patient population using a retrospective imaging analysis. By integrating MRI-based lesion classification with radiographic alignment profiles, this study seeks to elucidate potential biomechanical factors that influence the development and distribution of endplate lesions, thereby supporting more precise, mechanism-informed clinical decision-making.

## Methods

### Dataset

**Fig. 1:**
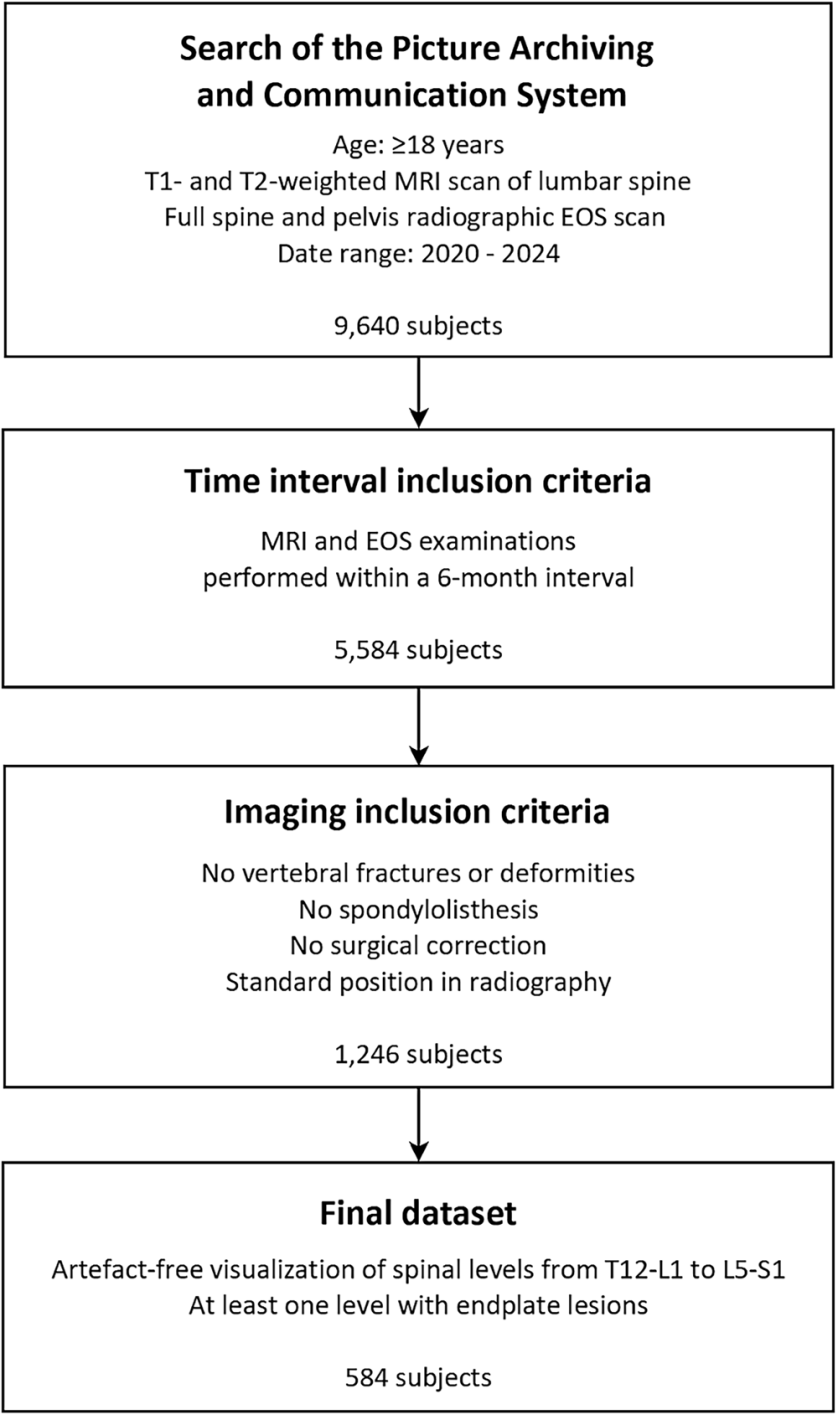
Flowchart illustrating the workflow for the identification of the evaluated dataset

A retrospective search of the Picture Archiving and Communication System of the IRCCS Galeazzi-Sant’Ambrogio Hospital (Milan, Italy) was performed on anonymized data acquired in the period 2020–2024 (Fig. 1). Subjects were included if they met the following criteria: age ≥ 18 years; availability of sagittal T1-weighted and T2-weighted MRI scans of the lumbar spine acquired on one of two 1.5-T systems (Avanto and Espree, Siemens Healthineers); and radiographic examination of the spine and pelvis obtained with the EOS system (EOS Imaging) allowing for the simultaneous acquisition of true size coronal and sagittal images in one-to-one scale avoiding vertical distortion [19–21]. Only cases with MRI and EOS examinations performed within a 6-month interval were retained.

The MRI and radiographic sagittal images were evaluated by a panel consisting of two experienced radiologists and one experienced spine surgeon. Subjects with vertebral fractures or deformities, spondylolisthesis, prior surgical correction, evident alterations of vertebral alignment within the thoracic and lumbar regions attributable to scoliosis, or non-standard positioning during radiography (*e.g*., seated posture or trunk flexion) were excluded. Only cases meeting the following criteria were retained: sagittal MRI slices with artifact-free visualization of spinal levels from T12–L1 to L5–S1, and sagittal radiographic images with a sufficient field of view to allow measurement of spinal alignment and spinopelvic parameters. Both T1-weighted and T2-weighted images (not fat‑suppressed sequences) were used for the radiological assessments. T1-weighted sequences are valuable for evaluating fatty infiltration, anatomical detail, and bone marrow adjacent to the endplate, whereas T2-weighted sequences, owing to their sensitivity to water content, are routinely employed in the assessment of general spinal pathology and are particularly useful for identifying fluid, edema, and other abnormalities.

### Radiological measurements

**Fig. 2:**
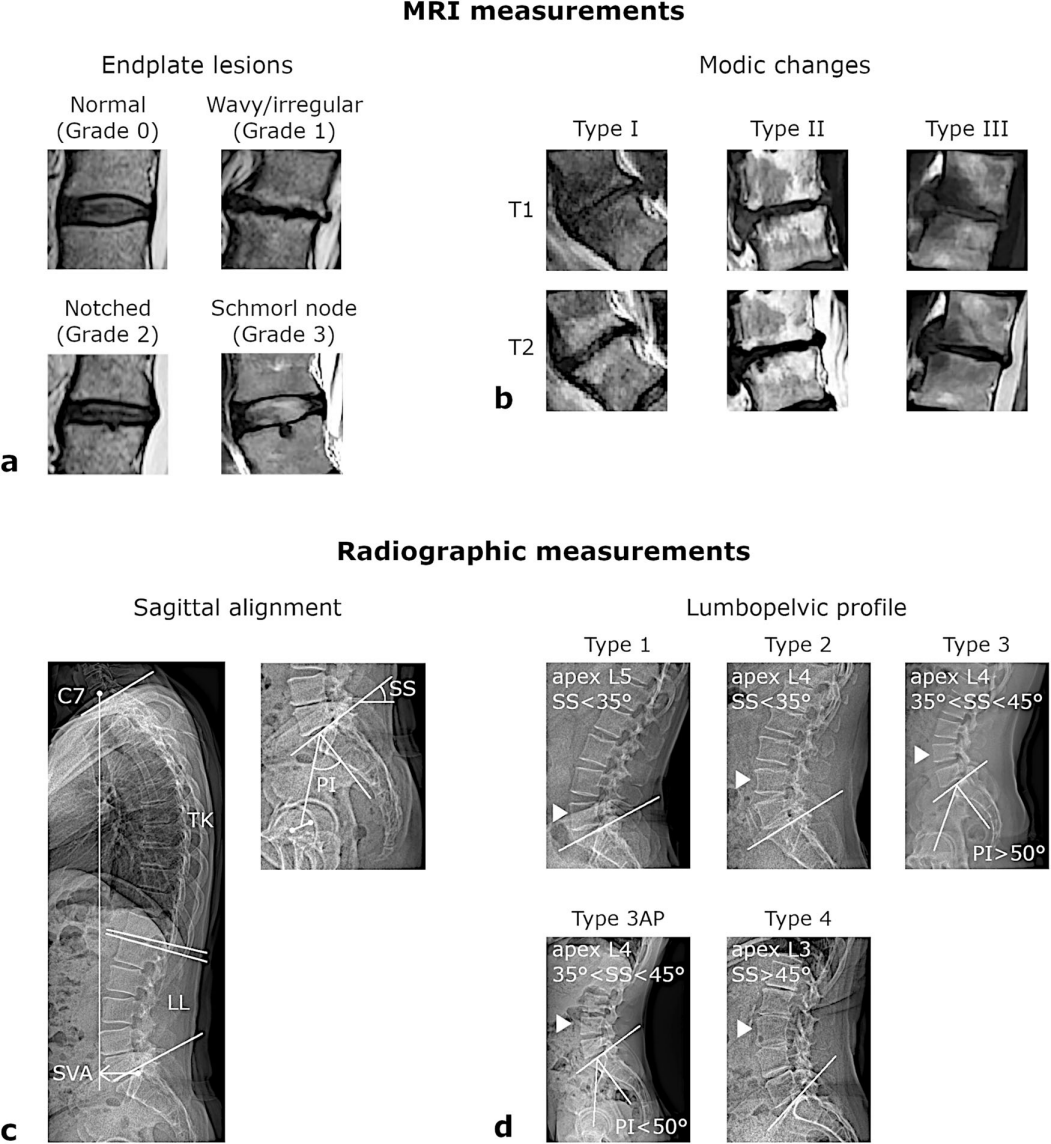
Upper panel: magnetic resonance imaging measurements, specifically endplate lesion types (**a**) and Modic changes (**b**) of intervertebral levels drawn from different subjects. Lower panel: radiographic measurements, illustrating spinal alignment parameters TK, LL, SS, PI, and SVA (**c**), and lumbopelvic profile types according to the Roussouly classification scheme (**d**). LL, Lumbar lordosis; PI, Pelvic incidence; SS, Sacral slope; SVA, Sagittal vertical axis; TK, Thoracic kyphosis

The dataset was randomly divided into three equally sized subsets, and the MRI and radiographic parameters for each subset were independently assessed by one rater from the pool. Endplate lesions at intervertebral levels from T12–L1 to L5–S1 were assessed on the MRI images according to a classification scheme based on shape [5]. The scoring system ranges from 0 to 3, reflecting increasing severity of endplate lesions, and is defined as follows (Fig. 2a):

- Grade 0 (normal) = no lesions are identifiable in sagittal MRI slices of the intervertebral space;
- Grade 1 (wavy/irregular) = no discrete lesions are visible within the intervertebral space, but at least one endplate exhibits an altered contour compared to the normal curvature of a healthy intervertebral space, appearing wavy or irregular;
- Grade 2 (notched) = a small focal lesion is visible in at least one sagittal MRI slice. The lesion has a V-shaped or circular configuration and may involve one or both endplates, suggesting localized defects or indentations;
- Grade 3 (Schmorl node) = a deep focal defect is observed in the vertebral endplate, characterized by smooth margins and a rounded appearance (Schmorl nodes correspond to protrusion of disc tissue through the endplate into the vertebral marrow).

The shape-based classification scheme categorizes endplate lesion severity at the intervertebral level using a single image slice, reflecting a progressive severity scale from grade 1 to grade 3, regardless of lesion extent. Because an intervertebral volume assessed across multiple slices may include more than one lesion type, including normal (grade 0) regions, the representative score for each spinal level was determined by assigning the highest severity grade observed across all sagittal slices (*e.g*., if grades 0, 2, and 3 were present, the final score for that level was 3). This approach ensures consistency with the underlying principle of the classification scheme. Only subjects with at least one spinal level exhibiting an endplate lesion were retained, whereas cases with a grade of 0 across all levels were excluded. Accordingly, the final dataset included 584 subjects (Fig. 1).

Four summarizing indices were designed to capture both the overall burden and the spatial distribution of endplate lesions within the lumbar spine. Specifically: (1) the sum of scores across all spinal levels, providing a global measure of lesion severity, integrating the cumulative morphological alterations along the lumbar region, regardless of location; (2) the difference between the sum of the three upper levels (T12–L1 to L2–L3) and the three lower levels (L3–L4 to L5–S1), quantifying the relative predominance of lesions along the cranio-caudal axis, enabling evaluation of whether endplate damage preferentially affects the upper or lower lumbar segments; (3) the number of levels with lesions (representative score > 0), indicating the extent of lesion dissemination, indicating how many intervertebral levels show any degree of morphological alteration, reflecting spatial spread rather than intensity; and (4) the difference in the number of affected levels between the upper and lower regions, characterizing the topographic distribution of lesions by identifying whether the pattern of involvement is more pronounced in the upper or lower lumbar spine.

For each spinal level, Modic changes were also assessed and classified into three types based on signal intensity patterns on T1- and T2-weighted MRI sequences (Fig. 2b) [22, 23]. Modic type I reflects edema and inflammation, appearing hypointense on T1 and hyperintense on T2. Modic type II corresponds to fatty replacement of bone marrow, appearing hyperintense on both T1 and T2. Modic type III represents subchondral bone sclerosis, appearing hypointense on both T1 and T2. Although varying forms of Modic changes are frequently observed in elderly individuals and in spine degeneration—sometimes involving only one side (anterior or posterior, cranial or caudal) or differing in extent—the classification in the present study was based solely on the presence of edema, independent of the side or extent. This choice was motivated by our aim to investigate the relationship between Modic changes and the presence and type of endplate lesions based on shape. Accordingly, coherence with the endplate lesion classification at the intervertebral level was required, as this classification relies only on the presence and shape of the lesion and does not account for whether the lesion is present on one or both endplates or its extent.

The following parameters of spinal alignment and lumbopelvic profile were manually measured on the radiographic images: thoracic kyphosis (TK), defined as the angle between the line connecting the upper endplate corners of T1 and the line connecting the lower endplate corners of T12; lumbar lordosis (LL), defined as the angle between the line connecting the upper endplate corners of L1 and the line connecting the lower endplate corners of L5; sacral slope (SS), defined as the angle between the line connecting the corners of the sacral plate and a horizontal reference line; pelvic incidence (PI), defined as the angle between the line perpendicular to the sacral plate at its midpoint and the line connecting this point to the center of the bicoxofemoral axis; and sagittal vertical axis (SVA), defined as the horizontal distance between a plumb line dropped from the centroid of C7 and the posterior corner of the sacral endplate, with positive values indicating that the line falls anterior to the posterior corner of the sacral endplate (Fig. 2c). LL was measured between L1 and L5 rather than between L1 and S1 (which coincides with sacral slope, SS) to differentiate the respective contributions of LL and SS in their association with the presence and severity of lesions. This approach avoids merging the two parameters into a single measure, which would provide a less specific description in the association analysis. The lumbopelvic profile was classified according to the four-type scheme proposed by Roussouly et al [14], based on the measured SS and visual assessment of the lumbar curve and apex position (Fig. 2d). The additional fifth category, type 3 with anteverted pelvis (type 3AP), was also considered, defined by PI < 50° [15]. Cases that could not be classified within these categories (*i.e*., those with a mismatch between SS value and apex position, or with absence of an apex due to a flat lumbar spine) were assigned to a sixth group, designated as ‘Other.’

### Parameter evaluation and statistical analysis

Differences in mean values between females and males were assessed using unpaired *t*-test or, in cases of non-normal distribution, the Wilcoxon rank-sum test. Differences in proportions were evaluated using a two-proportion z-test. Associations between variables were examined by correlation analysis. The correlation coefficient was computed using either Pearson’s (r) or Spearman’s (ρ) method, depending on data distribution. A value of 0 indicated no linear correlation, whereas -1 and 1 indicated complete negative and positive correlations, respectively. Statistical significance of Pearson’s correlation was tested using a two-tailed *t*-test, while the significance of Spearman’s correlation was assessed using a permutation test. To evaluate differences in parameter distributions across lumbopelvic profile categories, non-parametric approaches were adopted due to the generally non-normal distribution of variables. Specifically, the Kruskal–Wallis test was applied to assess differences in median values among the lumbopelvic profiles, followed by *post hoc* pairwise comparisons using the Tukey–Kramer method when significance was detected. Differences in frequencies of endplate lesion types and of severe cases across lumbopelvic profile were analyzed using the χ^2^ test, followed by *post hoc* pairwise comparisons using z-tests with Bonferroni correction where appropriate.

The predictive effects of demographic and alignment parameters (age, sex, lumbopelvic profile, TK, LL, PI, SS, and SVA) on the four regional endplate lesion scores (sum across levels, difference between upper and lower levels, number of severe levels, and difference in severe levels between upper and lower groups) were evaluated using a generalized multiple linear regression model. To reduce multicollinearity, parameters with correlation coefficients greater than 0.5 were not entered simultaneously. Rater was modeled as random effect. The significance of predictors was evaluated by testing regression coefficients using *t*-tests. For the lumbopelvic profile, significance was assessed by comparing the deviance of the full model with that of a reduced model excluding this factor, using a likelihood ratio test. For numerical predictors identified as significant, the strength of association with outcome scores was further quantified using partial Spearman correlation coefficients, which account for potential confounding from other input variables. The predictive effects of the same parameters on endplate lesion type (ordinal outcome) at each spinal level were evaluated using cumulative link mixed models. In these models, the above parameters and spinal level were included as fixed effects, while subject and rater were modeled as random effects. In addition, the predictive value of these parameters for the presence/absence of severe endplate lesions (binary outcome) was examined using generalized linear mixed-effects models with a binomial logistic outcome. Given the retrospective design of the study, which aimed to include the largest possible number of subjects based on availability in the institutional Picture Archiving and Communication System (PACS) system, no a priori power analysis was performed. Nonetheless, the final dataset was verified to meet statistical power requirements for the regression analysis. Specifically, the available sample size (584 subjects) was determined to be sufficient to detect a regression coefficient significantly different from zero in a multiple regression model with a small reference effect size (Cohen f² = 0.02), α = 0.05, and power = 0.92, for up to 20 predictors (G*Power software v3.1.9) [24].

All statistical tests adopted a significance level of α = 0.05. Analyses were performed using MATLAB software (v.R2024b, MathWorks Inc.), while cumulative link mixed model analyses were conducted in R (www.R-project.org) with the ordinal package.

## Results

Given the large number of evaluated parameters and performed comparisons, the results are divided and presented as follows: age, alignment parameters, and distribution of lumbopelvic profiles in the overall population, females, and males (Table 1); correlation coefficients between sagittal parameters (Table 2); distribution of endplate lesion types in relation to Modic changes and lumbopelvic profiles (Table 3); computed endplate lesion indices in relation to Modic changes and lumbopelvic profiles (Table 4); and predictive effects of demographic and alignment parameters on endplate lesion indices and types (Table 5). A total of 584 subjects (317 females, 54%; 267 males, 46%), aged 18–87 years (median: 56 years; interquartile range: 24 years), with a median interval of 0 days (interquartile range, 27 days) between MRI and radiographic examination, were evaluated (Table 1). The median age was slightly but significantly higher in females (60 years) compared with males (51 years, *p* < 0.001). Median values and ranges for spinal alignment parameters (TK, LL, SS, PI, and SVA) were similar between sexes and showed very weak correlations with age (absolute values < 0.3), except for SVA, which demonstrated a moderate positive correlation (ρ = 0.52 in females and ρ = 0.43 in males, with *p* < 0.001 in being statistically different from zero). The lumbopelvic profiles were distributed as follows: 126 (22%) cases type 1, 158 (27%) type 2, 112 (19%) type 3, 40 (7%) type 3AP, 60 (10%) type 4, and 88 (15%) unclassifiable cases grouped as ‘Other.’ The distribution across lumbopelvic types was similar between sexes, with the exception of a significantly higher proportion of males in type 3AP (9% *versus* 5% in females, *p* = 0.027). The median age was greater in females than in males, with significant differences observed in types 1 (*p* = 0.005), 2 (*p* = 0.034), 3 (*p* = 0.036), and Other (*p* = 0.003).

**Table 1:** Age, alignment parameters and distribution of lumbopelvic profile in the overall population, females, and males

	Demographic and alignment parameters			
	All	Females	Males	Correlation with age (females/males)
Subjects	584	317 (54%)	267 (46%)	-
Age (years)	56 (23), 18–87	60 (22), 18–87	51 (24), 18–87^#^	-
TK (°)	49 (17), 3–93	50 (17), 10–93	49 (17), 3–84	0.2*/0.02
LL (°)	37 (18), 1–85	39 (18), 1–75	36 (18), 1–85	-0.27*/-0.3*
SS (°)	34 (12), -3 to 68	33 (12), 1–54	34 (12), -3 to 68	-0.17*/-0.17*
PI (°)	50 (16), 25–91	50 (16), 25–91	50 (15), 25–86	0.11/0.11
SVA (cm)	0.85 (4.7), -7.5 to 17.5	0.6 (4.7), -6.3 to 15	1.1 (4.6), -7.5 to 17.5	0.52*/0.43*

	Lumbopelvic profile			
	All	Females	Males	Age in females/males median (IQR)
1	126 (22%)	73 (23%)	53 (20%)	61 (17)/53 (18)^#^
2	158 (27%)	81 (26%)	77 (29%)	59 (23)/53 (24)^#^
3	112 (19%)	62 (20%)	50 (19%)	59 (26)/52 (17)^#^
3AP	40 (7%)	15 (5%)	25 (9%)^#^	54 (19)/39 (28)
4	60 (10%)	29 (9%)	31 (12%)	49 (33)/44 (26)
Other	88 (15%)	57 (18%)	31 (12%)^#^	64 (21)/51 (24)^#^

Data reported as number of cases (percentage relative to the total number of subjects and within each sex group); median (interquartile range, IQR) and range; correlation coefficient

* Significantly different from zero (*p* < 0.05)

^#^ Significant difference in median value or proportion between females and males (*p* < 0.05).

**Table 2:** Correlation coefficient between sagittal parameters

	LL	SS	PI	SVA
TK	0.39*	0.05	0.08	0.05
LL	-	0.72*	0.48*	-0.29*
SS	-	-	0.67*	-0.12*
PI	-	-	-	0.17*

*LL* Lumbar lordosis, *PI* Pelvic incidence, *SS* Sacral slope, *SVA* Sagittal vertical axis, *TK* Thoracic kyphosis

* Correlation coefficient significantly different from zero (*p* < 0.05)

**Table 3:** Distribution of endplate lesion types

	Modic change					
	None	Type I	Type II	Type III		
Total levels (*n* = 3,504)	2,930	227	330	17		
Normal (*n* = 1,422)	1,380 (47%)	16 (7%)	24 (7%)	2 (12%)		
Wavy/irregular (*n* = 1,055)	767 (26%)	116 (51%)	163 (49%)	9 (53%)		
Notched (*n* = 686)	565 (19%)	45 (20%)	74 (22%)	2 (12%)		
Schmorl node (*n* = 341)	218 (7%)	50 (22%)	69 (21%)	4 (24%)		

	Lumbopelvic profile					
	1	2	3	3AP	4	Other
Total levels (*n* = 3,504)	756	948	672	240	360	528
Normal	28% (2, 3, 3AP, 4, Oth.)	37% (1, 3, 3AP, 4)	44% (1, 2, 3AP, 4)	50% (1, 2)	59% (1, 2, 3, Oth.)	41% (1, 4)
Wavy/irregular	27% (4)	30% (4)	25% (4)	22%	18% (1, 2, Oth.)	30% (4)
Notched	30% (3, 3AP, 4, Oth.)	24% (4)	21% (1, 4)	18% (1)	13% (1, 2, 3)	18% (1)
Schmorl node	16% (2, 3)	10% (1)	9% (1)	10%	10%	11%

	Subjects with severe cases					
	1	2	3	3AP	4	Other
Total subjects (*n* = 584)	126	158	112	40	60	88
Severe cases (*n* = 403)	103 (82%) (3, 3AP, 4)	113 (72%)	72 (64%) (1)	21 (53%) (1)	36 (60%) (1)	58 (66%)
Females, males (*n* = 218, 185)	58 (79%), 45 (85%)	59 (73%), 54 (70%)	43 (69%), 29 (58%)	7 (45%), 14 (56%)	14 (48%), 22 (71%)	37 (65%), 21 (68%)

Number of levels and percentage of each lesion relative to the total within each column category. From top to bottom: distribution by Modic change type; by lumbopelvic profile (with *post hoc* comparison results in brackets, *p* < 0.05); and number and percentage of subjects presenting at least one severe lesion (notched vertebra or Schmorl node), including the proportion of female and male subjects relative to the total number of females and males within each lumbopelvic profile

**Table 4:** Results for computed endplate lesion indices in the lumbar region

	Sagittal parameter						
Total subjects (*n* = 584)	TK	LL	SS	PI	SVA		
Score sum (all levels)	0	-0.28*	-0.22*	-0.13*	0.15*		
Score difference(upper *minus* lower levels)	0.06	0.02	0.0	0.0	0.01		
Levels with lesions	-0.01	-0.31*	-0.26*	-0.15*	0.21*		
Levels with lesions difference (upper-lower levels)	0.06	0.02	0.0	-0.02	0.0		

	Lumbopelvic profile (Females, Males)						
	All	1	2	3	3AP	4	Other
Score sum (all levels)	5 (5), 6 (6)	6 (5), 8 (5)	6 (4), 6 (6)	5 (5), 5 (5)	4 (6), 5 (6)	3 (4), 5 (6)^#^	5 (6), 5 (6)
Score difference (upper *minus* lower levels)	0 (3), 0 (3)	0 (3), 1 (3)	0 (3), 0 (3)	1 (3), 0 (3)	0 (4), 0 (3)	-1 (4), -1 (3)	0 (2), -1 (5)
Levels with lesions	3 (3), 4 (3)	4 (2), 5 (2)	4 (3), 4 (2)	3 (3), 3 (3)	3 (3), 3 (3)	2 (2), 3 (3)	4 (3), 3 (3)
Levels with lesions difference (upper *minus* lower levels)	0 (2), 0 (2)	0 (2), 0 (1)	0 (1), 0 (2)	0 (2), 0 (2)	0 (3), 0 (2)	-1 (1), 0 (2)	0 (1), -1 (1)

From top to bottom: correlation coefficients between endplate lesion indices and sagittal parameters; median (interquartile range) of endplate lesion indices in females and males by lumbopelvic profile type

*LL* Lumbar lordosis, *PI* Pelvic incidence, *SS* Sacral slope, *SVA* Sagittal vertical axis, *TK* Thoracic kyphosis

* Coefficient significantly different from zero (*p* < 0.05)

^#^ Significant difference between females and males (*p* < 0.05) in the considered lumbopelvic profile type

**Table 5:** Predictive effects of demographic and alignment parameters on endplate lesion indices and types

	Prediction of endplate lesion indices						
(*N* = 584 subjects)	Age	Sex	TK	LL	PI	SVA	
Score sum (all levels)							
Estimate*	Positive (0.26)	Positive (n.a.)	-	Negative (-0.16)	-	-	
Score difference (upper *minus* lower levels)							
Estimate*	-	-	-	Positive (0.02)	-	-	
Levels with lesions							
Estimate*	Positive (0.26)	-	-	Negative (-0.14)	-	-	
Levels with lesions difference (upper *minus* lower levels)							
Estimate*	-	Positive (n.a.)	-	Positive (0.02)	-	-	

	Prediction of endplate lesion type						
(*N* = 3,504 levels)	Age	Sex	TK	LL	PI	SVA	Spinal level
Endplate lesion type							
Estimate^#^	Positive	Positive	-	Negative	-	-	Negative
Presence/absence of severe lesion							
Estimate^#^	Positive	-	-	Negative	-	Negative	Negative

From top to bottom: effects of parameter variables (age, sex, TK, LL, PI, SVA) on computed endplate lesion indices in the lumbar region, with partial correlation coefficients in brackets for numerical variables found to be significant predictors; effects of the same predictors and spinal level on endplate lesion type, and on the presence or absence of severe lesions

*LL* Lumbar lordosis, *PI* Pelvic incidence, *SS* Sacral slope, *SVA* Sagittal vertical axis, *TK* Thoracic kyphosis

* A positive estimate value in the Sex variable indicates a larger increasing effect by males compared to females (*p* < 0.05). The *p*-values for the estimates were tested for significance using a *t*-test. n.a. indicates non-applicable correlation. “-” indicates a non-significant estimate

^#^ A positive estimate value in the Sex variable indicates a more severe lesion (or the presence of a severe lesion) in males compared with females (*p*-value < 0.05). A negative value for the spinal levels variable indicates a more severe lesion (or the presence of a severe lesion) in the upper levels

**Fig. 3:**
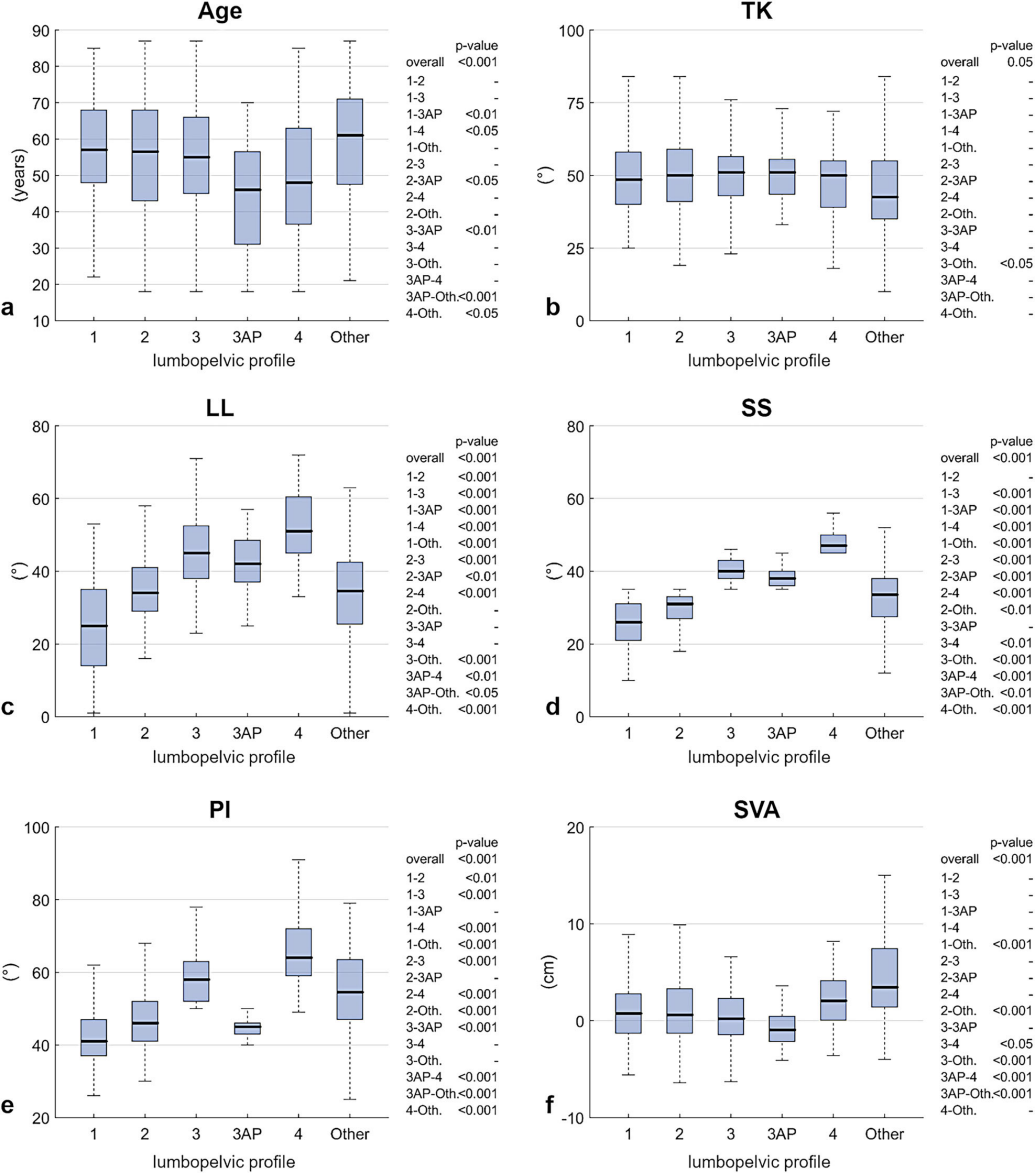
Distribution of demographic and sagittal alignment parameters across lumbopelvic types: age (**a**), TK (**b**), LL (**c**), SS (**d**), PI (**e**), and SVA (**f**). The *p*-values for overall differences in medians across lumbopelvic types, and *post hoc* comparisons when significant, are reported. LL, Lumbar lordosis; PI, Pelvic incidence; SS, Sacral slope; SVA, Sagittal vertical axis; TK, Thoracic kyphosis

Correlations among radiographic parameters were generally weak (Table 2), except for moderate-to-strong correlations between SS and PI (ρ = 0.67, *p* < 0.001), and between SS and LL (0.72, *p* < 0.001). Overall, the median age was lower in type 3AP and type 4 (below 50 years) compared with the other lumbopelvic types (Fig. 3a). Regarding spinal alignment parameters, TK was comparable across groups (Fig. 3b), whereas SVA showed higher positive values in groups 4 and Other (Fig. 3f). The distribution of the remaining parameters (LL, SS, and PI) varied according to the predefined ranges used to classify lumbopelvic types under the Roussouly scheme.

**Fig. 4:**
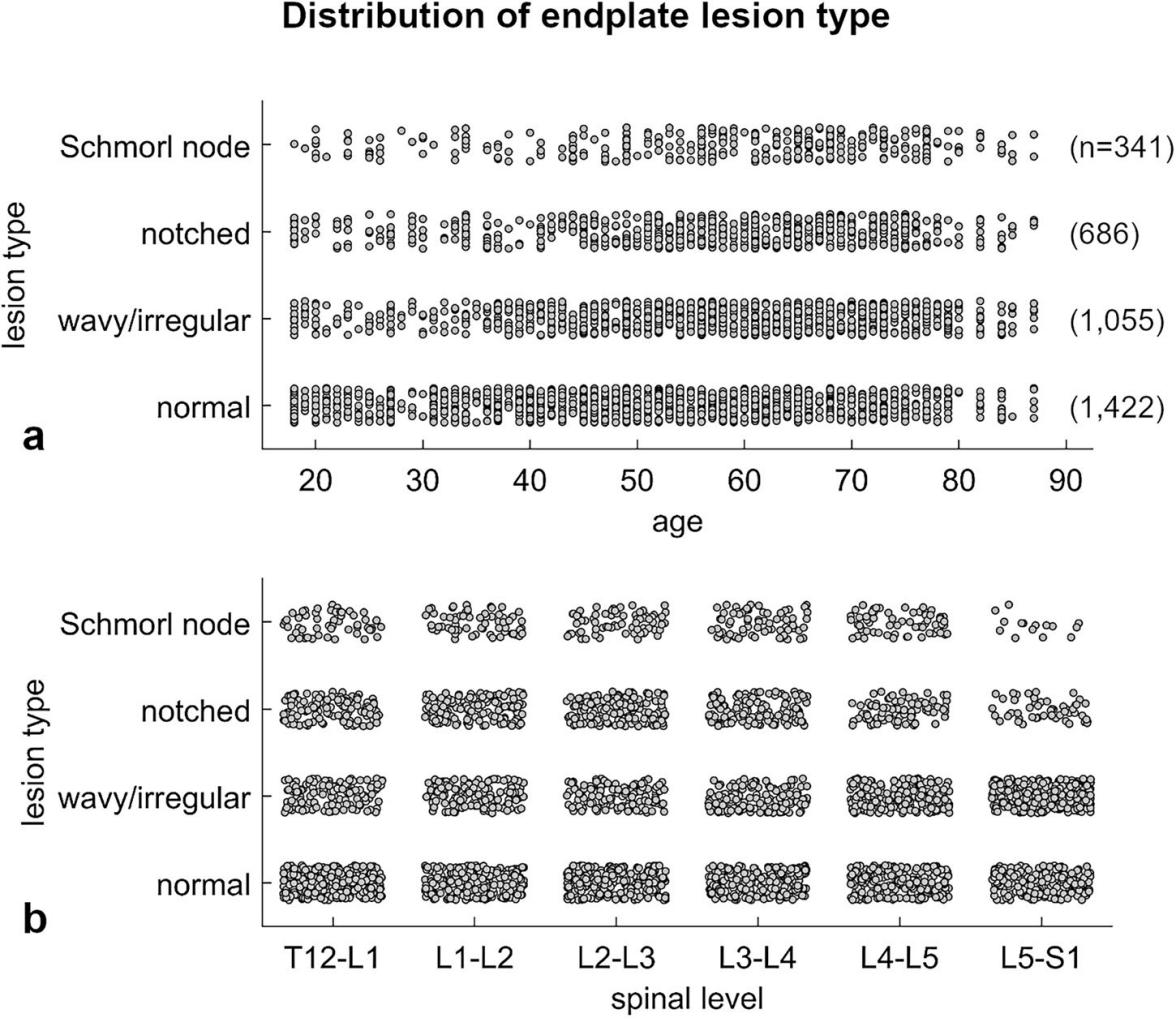
Scatter plots showing the distribution of endplate lesion types in relation to age (**a**) and spinal level (**b**)

Of the 3,504 spinal levels analyzed, endplate lesion types were distributed as follows: 1,422 (40%) normal, 1,055 (30%) wavy/irregular, 686 (20%) notched, and 341 (10%) Schmorl nodes (Table 3 and Fig. 4). The distribution of lesion types by age and spinal level showed that more severe lesions (notched and Schmorl nodes) became more prevalent with increasing age (Fig. 4a), and were slightly less prevalent at lower spinal levels (Fig. 4b). Regarding Modic changes, 2,930 levels (84%) showed no changes, while 227 (6%) were classified as Type I, 330 (9%) as Type II, and 17 (< 1%) as Type III (Table 3). Among Modic change types, the distribution of endplate lesion types was similar, with wavy/irregular lesions being the most prevalent (49–53% of levels), followed by notched and Schmorl node lesions, which were less frequent (12–24% of levels). Within lumbopelvic types, normal endplates were more prevalent in type 3AP and type 4 (50% and 59%, respectively) compared with the other types (28–44%), with correspondingly lower percentages of endplate lesions. The proportion of subjects with at least one level classified as severe was slightly lower in type 3AP (53%) and type 4 (60%) compared with the other types (64–82%), with comparable prevalence between females and males.

**Fig. 5:**
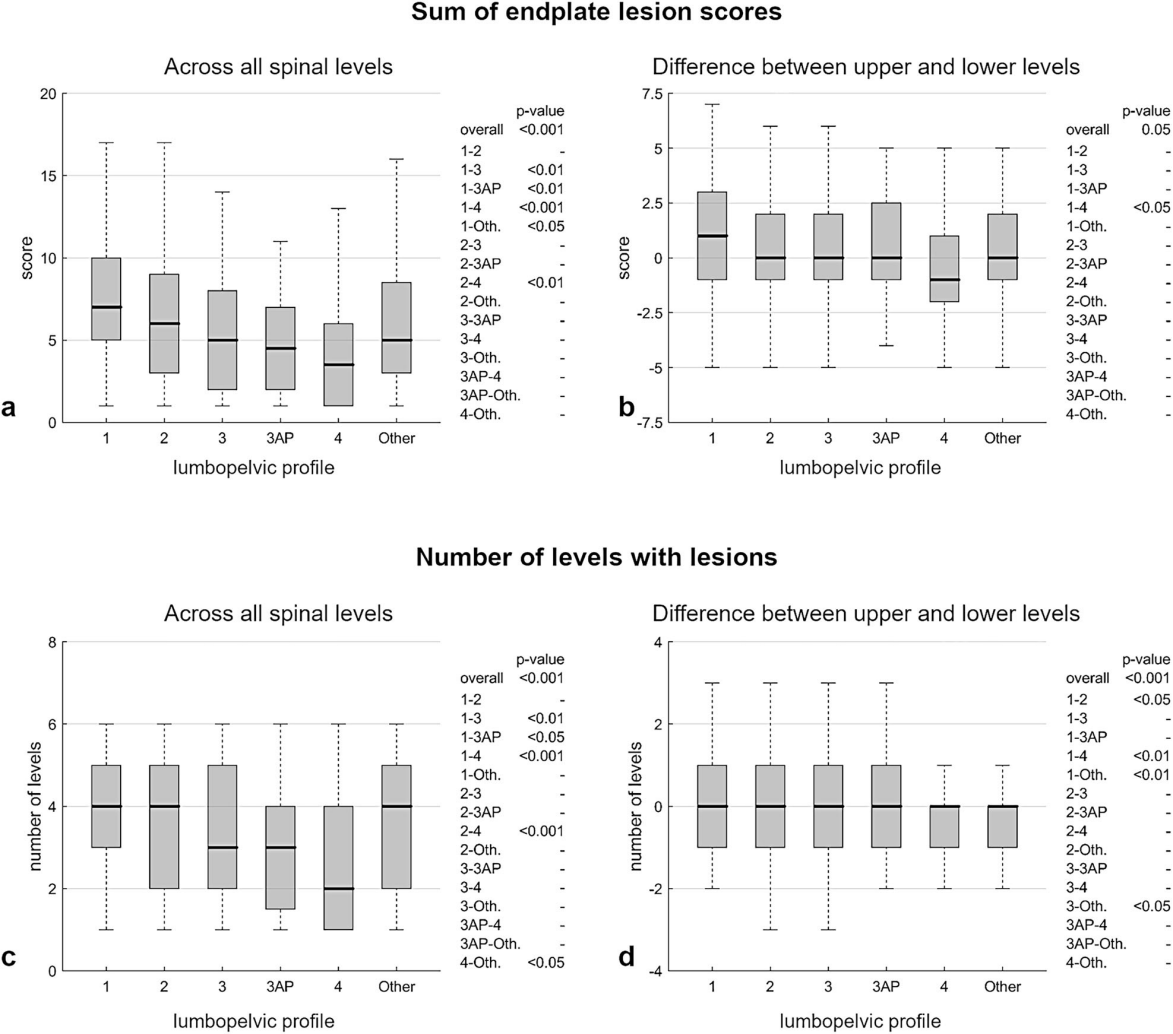
Distribution of endplate lesion computed indices in the lumbar region by lumbopelvic type: sum of scores across spinal levels (**a**); difference between upper and lower levels (**b**); number of levels with lesions (**c**); and difference in the number of affected levels between upper and lower regions (**d**). The *p*-values for overall differences in medians across lumbopelvic types, and *post hoc* comparisons when significant, are reported

Regarding the computed endplate lesion indices in the lumbar region, correlations with sagittal parameters were weak (absolute values < 0.29) for both the total score across levels and the number of levels with lesions (Table 4), and negligible for the differences in score or in the number of levels with lesions between upper and lower lumbar segments. Across lumbopelvic profile types, the median values of total score and number of levels with lesions were lower in type 3, 3AP, and 4 (Fig. 5a, c), whereas similar values were observed for the differences between upper and lower lumbar levels (Fig. 5b, d). Median values were generally statistically comparable between females and males (Table 4), with only one significant difference observed for the score sum in type 4 (3 *versus* 5 in females and males, respectively; *p* = 0.023).

When assessing the predictive value of demographic and alignment parameters on computed lesion indices, SS was excluded to reduce multicollinearity, given its strong correlation with LL and PI (Table 2). Among the remaining parameters, age, sex, and LL were significantly associated with both the total score and the number of levels with lesions, although correlations were weak (absolute values < 0.27, Table 5). Age and LL were also found to be associated with differences in scores and levels with lesions between upper and lower levels. In models evaluating the effects of demographic and alignment parameters together with spinal level on endplate lesion type, age, sex, LL, and spinal level were significantly associated with lesion type, whereas age, LL, SVA, and spinal level were significantly associated with the presence or absence of severe lesions. The lumbopelvic profile was generally not found to be a significant predictor in the regression models.

## Discussion

This is the first study to evaluate the association between the presence and distribution of endplate lesions, classified by shape, and spine sagittal alignment characteristics in a selected adult patient population. In contrast to previous studies that evaluated only the prevalence of endplate lesions, typically based on supine MRI scans [1, 2, 6–8, 12, 13], the present study enabled assessment of their association with spinal alignment parameters under upright loading conditions using radiographic examination (Fig. 2). This was made possible by exploiting a retrospective dataset of subjects who underwent both examinations within a 6-month interval, with 52% examined on the same day and the overall cohort showing a median interval of 0 days between examinations (interquartile range, 27 days).

With respect to the distribution of lumbopelvic profile, type 1 (22% of cases, Table 1) and type 2 (27%) were more prevalent than in the healthy younger population (aged 18–48 years) reported in previous studies (12% and 22%, respectively) [15]. In contrast, type 3 (19%), type 3AP (7%), and type 4 (10%) were less prevalent compared to the corresponding values in the healthy cohort (30%, 16%, and 20%, respectively). This discrepancy may be explained by the older age of the select population under study relative to the healthy control group. In elderly individuals, specific postural adaptations are known to occur: the lower lumbar spine tends to preserve its lordosis, whereas the central lumbar segments often flatten. To counterbalance degenerative spinal changes, compensatory mechanisms such as pelvic retroversion, knee flexion, and pelvic shift are activated during aging. These adaptations progressively reduce sacral slope (SS) and lumbar lordosis (LL)—parameters that are intrinsically lower in types 1 and 2—leading over time to increased pelvic retroversion and a more anteriorly imbalanced posture [25–29]. This is supported by our findings, which show a lower median age in type 3AP and type 4 (Fig. 3a), as well as a moderate positive correlation between SVA and age (0.52 and 0.43 for females and males, respectively; Table 1). The median PI was confirmed to be lower in types 1 and 2 compared with types 3 and 4 (Fig. 3e), consistent with findings in healthy younger populations [15]. However, since this was not a longitudinal study, no conclusions can be drawn regarding potential minor variations in PI—possibly related, for instance, to limited mobility of the sacroiliac joint—in association with age or lumbopelvic profile classification. Consequently, we cannot contribute to the ongoing debate in the literature on whether PI is entirely fixed or may have small variations over time [30–34]. It is noteworthy that 15% of cases were categorized as Other, as they could not be classified according to any of the Roussouly types. This occurred when no correspondence was observed between SS and the location of the lumbar curve apex, or in cases where curve flattening precluded identification of an apex vertebra. Specifically, this group comprised 53 cases with SS < 35° and apex at L3 or a flat curve; 26 cases with 35° < SS < 45° and apex at L3; and 9 cases with SS > 45° and apex at L4. This percentage of non-categorizable cases is consistent with our previous study on an elderly cohort of 154 asymptomatic subjects (aged 61–89 years), in which 16% of individuals could not be classified into any of the established lumbopelvic types [29]. This finding confirms the need for caution and highlights certain limitations in applying the Roussouly classification to elderly populations, whether asymptomatic or orthopedic. The results for these non-categorizable cases generally show median values that approximate the average of the categorized profiles. This finding was anticipated and can be explained by the fact that the Other group encompasses and mixes features of the other profiles that could not be classified due to the absence of a strict combination of sacral slope and lumbar curve apex as defined by the Roussouly classification.

Regarding the prevalence of endplate lesions, an increased frequency of severe lesions (notched and Schmorl node types) with advancing age was confirmed (Fig. 4a) [1, 3, 5, 7], particularly up to around 75 years, whereas the prevalence of all lesion types decreased thereafter due to the limited number of subjects and spinal levels available for analysis. Conversely, the association with concomitant alterations, such as Modic changes, was only partially corroborated [8, 12, 13]. Across the evaluated spinal levels, 84% showed no Modic changes; however, endplate lesions were identified in 53% of these levels: 26% wavy/irregular, 19% notched, and 8% Schmorl node (Table 3). Among levels exhibiting Modic changes (16% of total), wavy/irregular lesions were most prevalent (49–53%), followed by notched and Schmorl node lesions, which were less frequent (12–22%). These findings indicate a weak-to-moderate association between endplate lesions and the presence of Modic changes, corroborating previous studies that suggest endplate lesions may serve as initiating factors in the development of subsequent pathological processes [13, 35].

As regards the distribution of endplate lesions across lumbopelvic profiles, the prevalence of wavy/irregular and notched lesions was moderately lower in types 3AP, 3, and 4 (13–25%) compared with types 1 and 2 (24–30%). Furthermore, the proportion of subjects with at least one spinal level affected by a severe lesion was higher in types 1 and 2 (72–82%) than in the other profiles (53–64%), with no statistically significant difference between females and males. This trend was confirmed when assessing computed lesion indices in the lumbar region, namely the sum of endplate lesion scores (Fig. 5a) and the number of affected levels (Fig. 5c). The median value of the total score tended to decrease progressively from profile 1 to 4, while the number of affected levels was moderately lower in profiles 3, 3AP, and 4. By contrast, indices reflecting differences between upper and lower spinal levels showed comparable values across profiles (Fig. 5b, d). Results were also consistent between females and males (Table 4).

These findings are consistent with those of a recent study on 150 Chinese patients with osteoporosis (mean age 61 ± 7 years) [11], which reported higher lumbar endplate lesion scores in profiles 1 and 2—assessed using a lesion extension–based classification [10]—compared with profiles 3 and 4. In contrast, no significant differences were observed across lumbopelvic profiles in an age-matched control group of 150 Chinese individuals, supporting the hypothesis that profiles characterized by a lower lumbar arc and apex vertebra located in the lower spine confer less advantageous spinal load transfer at the endplate level, thereby facilitating lesion development in older individuals with pathological conditions.

When evaluating the predictive effect of age and radiographic parameters on endplate lesion indices, significant associations were observed with age, sex, and LL (Table 5). Specifically, increasing age was associated with more severe lesion scores and with a greater number of affected levels; slightly higher values were observed in males, in agreement with previous findings [6]. Lower LL was associated with a higher prevalence of lesions, further confirming that flatter lumbar alignment predisposes to endplate damage, most likely due to altered load transfer patterns. An association was also observed between LL and difference indices (reflecting discrepancies between upper and lower spinal levels), suggesting a greater presence of lesions in upper levels with larger LL. However, partial correlations between these indices and LL were negligible, indicating limited support for this finding. Regarding the prediction of lesion type and the presence of severe lesions, the indices confirmed the same associations, with the additional finding that spinal level was negatively associated, indicating more severe lesions in the upper lumbar levels. Nevertheless, it should be emphasized that, despite reaching statistical significance, the correlations between the evaluated variables and endplate lesion indices were generally weak (absolute values < 0.26). This finding is consistent with the weak direct correlations observed between lesion indices and sagittal parameters (absolute values < 0.29; see Table 4). Furthermore, the lumbopelvic profile was not identified as a significant predictor in the regression models, suggesting that classification according to the Roussouly scheme is not directly associated with the presence or type of lesion in the studied population.

Overall, our findings indicate that endplate lesions were more prevalent and severe in spinal profiles characterized by lower SS and reduced LL, suggesting a significant pathophysiological interplay between the vertebral endplate, intervertebral disc, and overall spinal alignment. The vertebral endplate serves as a critical interface for load transmission and nutrient exchange to the disc. When sagittal alignment is less favorable—such as in spines with attenuated lordosis—the mechanical load distribution becomes suboptimal, concentrating stress on the endplate rather than dispersing it evenly across the disc. This maladaptive loading may predispose the endplate to micro damage, sclerosis, and inflammatory changes, which in turn compromise disc nutrition and accelerate degenerative processes. Progressive disc height loss and vertebral remodeling further exacerbate sagittal imbalance, perpetuating a self-reinforcing cycle of structural deterioration. These observations support the hypothesis that biomechanical factors, particularly those related to global alignment, play a pivotal role in the development of endplate lesions in aging spines. Clinically, this relationship underscores the importance of considering sagittal profile in both preventive strategies and therapeutic decision-making, as endplate integrity is not only a marker of disc health but also a determinant of spinal stability and pain generation. Future research should explore whether targeted interventions aimed at optimizing load transfer—through posture correction, rehabilitation, or surgical alignment—could mitigate endplate damage and slow the degenerative cascade.

Regarding the limitations of the study, the following specific considerations should be noted. No clinical or radiological reports were filtered or available during the retrospective search; therefore, the specific clinical indications for MRI and EOS acquisition could not be determined. Consequently, patients may have undergone examinations for a range of standard clinical purposes, including diagnostic assessment, follow-up or monitoring of degenerative changes, or preoperative planning. Moreover, no information regarding pain intensity, functional status, or disability was available for inclusion in the present analysis. The observed prevalence of endplate lesions does not reflect that of the overall orthopedic population, as only patients who met all inclusion criteria and presented with at least one lumbar level affected by lesions were included (Fig. 1). Consequently, direct comparison with studies conducted on the general population is not possible. This selection was necessary to accurately assess the relationship between the prevalence and severity of lesions and spinal alignment. Furthermore, lesion grading, Modic changes, and the measurement of radiographic parameters were performed by a pool of two experienced radiologists and one experienced spine surgeon. The inter-rater reliability in measuring the radiological parameters was not specifically evaluated in the present study. This is because high inter- and intra-observer reliability of endplate lesion classification, Modic changes, and other radiological parameters has already been demonstrated in the literature [5, 36–38]. Moreover, the raters in this study had comparable levels of expertise in terms of years of practice, limiting potential differences related to experience. In the data analysis, to mitigate potential bias, the ‘rater’ was modeled as a random-effect variable in the regression analysis, which did not reveal any significant impact. Furthermore, verification of the results within subgroups of the complete dataset—each independently evaluated by one of the three raters—did not show any substantial differences. The retrospective and cross-sectional design of the study does not allow investigation of temporal changes in lesion severity, nor assessment of how the defined categories of endplate lesions may represent a continuum of the same pathology. In this regard, it should also be acknowledged, as reported in the literature, that wavy or notched endplate alterations can be observed even in asymptomatic younger individuals or pediatric populations. Further analyses incorporating radiological parameters of disc condition—such as disc degeneration grading and canal narrowing, which are known to be associated with spinal alignment [39–41] and the presence of endplate lesions [35, 42–45]—were not performed, as these fall outside the specific aim of the present study, which was to address the gap concerning how spinal alignment parameters influence the type and distribution of lesions across lumbar levels. From a pathophysiological perspective, the vertebral endplate and intervertebral disc constitute a functional unit; accordingly, no conclusions can be drawn about either component individually, nor about their interplay in relation to alignment, based on the current data. Lastly, although subjects with evident alterations in vertebral alignment within the thoracic and lumbar regions attributable to scoliosis were excluded from the analysis to ensure precise measurement and interpretation of endplate parameters, coronal radiographic images were not evaluated to quantify scoliosis severity. Therefore, the presence of mild, unrecognized scoliosis could not be entirely excluded.

In conclusion, endplate lesions were more prevalent and severe in profiles characterized by lower sacral slope and lumbar lordosis, supporting the hypothesis that less favorable load transfer at the endplate level predisposes to lesion development in aging spines. Although sagittal parameters and lumbopelvic profiles influence lesion distribution, their predictive value appears limited, underscoring the complex and multifactorial nature of endplate degeneration.

## Data Availability

The datasets used and/or analyzed during the current study are available from the corresponding author on reasonable request.

## Abbreviations

LL: Lumbar lordosis
MRI: Magnetic resonance imaging
PI: Pelvic incidence
SS: Sacral slope
SVA: Sagittal vertical axis
TK: Thoracic kyphosis
